# Femoral Stem Design Influences Early Periprosthetic Fracture Risk Following Total Hip Arthroplasty

**DOI:** 10.1016/j.artd.2026.102193

**Published:** 2026-10-31

**Authors:** Alexandra N. Krez, Sean D. Toomey, Kyle C. Swanson, Carl L. Herndon, Jonathan R. Danoff

**Affiliations:** a Department of Orthopaedic Surgery, Northwell Health, North Shore University Hospital, Manhasset, NY, USA; b Department of Orthopedic Surgery, PLLC, Swedish Orthopedic Institute, Seattle, WA, USA; c The Orthopaedic & Fracture Clinic, Mankato, MN, USA; d Department of Orthopedic Surgery, Columbia University Medical Center, New York, NY, USA

**Keywords:** Periprosthetic fracture, Triple-taper metaphyseal stem, Collar, Stem morphology

## Abstract

**Background:**

Periprosthetic femur fracture (PFF) is a significant complication following total hip arthroplasty (THA). Modern collared, triple-taper metaphyseal-filling stems may reduce fracture risk. This study compared a triple-taper metaphyseal stem with other cementless designs to assess early PFF risk, fracture risk by age and bone quality, and the impact of collar position.

**Methods:**

This retrospective study analyzed 774 primary cementless THAs, including triple-taper metaphyseal (n = 311), single-wedge (n = 294), double-wedge (n = 116), and gradual taper/metadiaphyseal-filling (n = 53) stems. Triple-taper stems were implanted through a prospective multicenter trial, while comparison stems were from a single health system. Patients were stratified by stem design, age, Dorr classification, and collar position. The primary outcome was 90-day PFF.

**Results:**

PFF occurred in 53 patients (6.8%), including 15 (1.9%) postoperative fractures requiring reoperation. Patients with PFF were older (*P* < .001) and predominantly female (*P* = .02). Triple-taper stems demonstrated lower PFF risk than other designs (odds ratio 0.20; 95% confidence interval, 0.08–0.48; *P* < .001). PFF rates were 1.9% for triple-taper, 7.8% for single-wedge, 14.7% for double-wedge, and 13.2% for gradual taper stems (all *P* < .001). Differences persisted across age groups and Dorr A/B femurs. Proud collar positioning increased PFF risk (6.3% vs 0.8%; *P* = .02).

**Conclusions:**

Collared, triple-taper metaphyseal stems were associated with lower early PFF risk across patient ages and bone quality profiles. Seating the collar on the calcar further reduced fracture incidence.

## Introduction

Periprosthetic femur fracture (PFF) is one of the most debilitating and costly complications following total hip arthroplasty (THA), often necessitating reoperation, prolonged recovery, and extended rehabilitation [1], [2], [3]. PFF are associated with a 3% 90-day mortality rate and minor complications occur in approximately 59% of these cases [4]. Further, functional outcomes are often compromised in 20% of patients, despite fracture healing [5]. As the number of primary THAs continues to rise globally [6], understanding and mitigating the risk of early mechanical failure secondary to periprosthetic proximal femur fracture becomes increasingly important. Several patient-related factors such as increased age [7], [8], female sex [7], [9], and diminished bone quality as classified by Dorr classification [9] are well-established contributors to early PFF risk.

Femoral stem geometry and the presence of a collar influence the risk of PFF. In a recent registry analysis of over 230,000 cementless THAs in patients over 65 years old, single- and double-wedge stem designs were associated with nearly three-times increased risk of PFF compared to implants with specific geometric features, such as metaphyseal tapering and proximal calcar support. These designs may reduce micromotion and enhance femoral component stability [10]. More recently, Neitzke et al. [11] demonstrated a threefold reduction in early PFF risk with a modern triple-tapered, noncemented, collared stem using American Joint Replacement Registry data, with lower fracture rates than other cementless stem designs and rates comparable to cemented fixation. Collectively, these findings suggest that triple-tapered metaphyseal stem with collared designs may provide enhance early mechanical stability and represent safer cementless option for reducing PFF risk.

The primary goal of this study was to determine if triple-taper metaphyseal stems are independently associated with risk of early PFF (within 90 days) after cementless THA. Secondarily, we sought to determine within the cohort of triple-taper metaphyseal stems, the influence of collar position (proud vs on the calcar) on PFF risk. We hypothesized that triple-taper femoral components with a collar will have a lower periprosthetic femur fracture risk than other geometric shaped stems.

## Material and methods

This study obtained approval by the local Institutional Review Board (IRB 25-0282), femoral stems were classified and adapted according to the Kheir and Chen system [12]. The study population included 311 patients undergoing THA with 1 type of triple-taper metaphyseal stem (Insignia, Stryker, Mahwah, NJ) between February 1, 2022 and April 30, 2024 as part of a prospective, multicenter clinical trial. The experimental cohort was compared to a previously published independent cohort from a tertiary academic medical center based in an urban environment between January 1, 2014 and February 28, 2019, comprising type 2 (single-wedge; n = 294), type 3 (double-wedge; n = 116), and type 4B (gradual taper rectangular metaphyseal-filling rectangular; n = 53) stems (Table 1) [9]. A femoral collar was present on both the triple-taper metaphyseal and type 4B gradual taper/metadiaphyseal-filling stem designs. A minimum postoperative follow-up of 90 days was required for inclusion. Patients undergoing surgery for a diagnosis of THA revision or oncological diagnosis or with incomplete follow-up data were excluded.

**Table 1:** Femoral stem types.

Stem type	Brand and model	n (%)
2: Single wedge	Accolade II	124 (16.0%)
	Anthology	73 (9.4%)
	ML taper	97 (12.5%)
3: Double wedge	Synergy	111 (14.3%)
	Secure-Fit	5 (6.5%)
4A: Triple-taper metaphyseal	Insignia	311 (40.2%)
4B: Gradual taper /metadiaphyseal filling	Polarstem	53 (6.8%)

Demographic and implant-related variables were available from blinded records of both cohorts including age at surgery, sex, laterality, body mass index (BMI), preoperative diagnosis, surgical approach, and stem type. A blinded, independent orthopaedic surgeon reviewed all radiographs and determined bone quality as measured by Dorr classification (A, B, or C) using the preoperative radiographs obtained prior to surgery [13]. Collar position was measured for the triple-taper metaphyseal cohort only, as distance of the collar relative to the calcar on postoperative anteroposterior pelvis radiographs taken at 3 months from surgery. Intraoperative and postoperative PFFs were recorded. Postoperative fractures were defined as femoral fractures occurring intraoperatively and within 90 days postoperatively.

### Data analyses

Independent variables included, age, sex, Dorr ratio, and implant type. The dependent variable was intraoperative or early postoperative PFF. Categorical variables were compared using *Chi*-square tests, and continuous variables using *t*-tests or ANOVA as appropriate. A multivariable logistic regression model was constructed to evaluate the association between stem type and early PFF, adjusting for age, sex, and bone quality. For comparisons evaluating the impact of collar presence, *P* values were calculated using Fisher's exact test. Odds ratios (ORs) are presented with 95% confidence intervals (CIs), with the triple-taper metaphyseal stem serving as the reference group. Subgroup analysis of collar position was limited to the triple-taper metaphyseal stem cohort. Statistical significance was defined as *P* < .05. A post hoc power analysis was conducted to evaluate the adequacy of the sample size for detecting an association via *Chi*-square test of independence. The observed effect size was calculated as a total sample size of *N* = 774 and a significance level of α = 0.05, the achieved statistical power was 1.00, indicating that the analysis was fully powered to detect the observed effect. All statistical analyses were conducted using R version 4.3.0 (R Foundation for Statistical Computing, Vienna, Austria).

## Results

Of the 774 patients who underwent primary THA, the mean age was 65.5 ± 11 years and mean BMI was 27.7 ± 5.3 kg/m^2^. The most common indication for surgery was osteoarthritis (89.7%), followed by avascular necrosis (7.4%), femoral neck fracture (1.9%), and rheumatoid arthritis (0.8%). The most common Dorr category was Dorr A (64.9%). Patients who received triple-taper metaphyseal stem underwent either direct anterior (n = 205) posterior (n = 103) or anterolateral (n = 3) approach THA (Table 2) by fellowship trained arthroplasty surgeons from one of 11 different medical centers. All patients in the comparison cohort (n = 463) underwent THA via an anterolateral approach by one of three fellowship trained arthroplasty surgeons at a single medical center.

**Table 2:** Patient demographics and clinical characteristics by occurrence of PFF within 90 days following cementless THA.

Patient demographics	Total	No fracture	Fracture	*P* value
	(n = 774)	(n = 721)	(n = 53)	
Gender, n (%)				**.02**
Male	331 (42.8%)	317 (44.0%)	14 (26.4%)	
Female	443 (57.2%)	404 (56.0%)	39 (73.6%)	
Age (y), mean (SD)	65.5 (11.0)	65.1 (11.0)	70.5 (9.5)	**<.001**
Diagnosis, n (%)				.33
Osteoarthritis	694 (89.7%)	648 (89.9%)	46 (86.8%)	
Avascular necrosis	57 (7.4%)	53 (7.4%)	4 (7.5%)	
Rheumatoid arthritis	6 (0.8%)	6 (0.8%)	0 (0.0%)	
Femoral neck fracture	15 (2.0%)	12 (1.7%)	3 (5.7%)	
Femoral head fracture	1 (0.1%)	1 (0.1%)	0 (0.0%)	
Hemiplegic cerebral palsy	1 (0.1%)	1 (0.1%)	0 (0.0%)	
Laterality, n (%)				.51
Left	368 (47.5%)	340 (47.2%)	28 (52.8%)	
Right	406 (52.5%)	381 (52.8%)	25 (47.2%)	
Body mass index (kg/m2), mean (SD)	27.7 (5.3)	27.7 (5.3)	27.4 (5.1)	.71
Dorr classification, n (%)				.42
A	502 (64.9%)	472 (65.5%)	30 (56.6%)	
B	230 (29.7%)	209 (30.0%)	21 (39.6%)	
C	16 (2.1%)	15 (2.1%)	1 (1.9%)	
Surgical approach, n (%)				**<.001**
Anterolateral	466 (60.2%)	419 (58.1%)	47 (88.7%)	
Direct anterior	205 (26.5%)	203 (28.2%)	2 (3.8%)	
Posterolateral	103 (13.3%)	99 (13.7%)	4 (7.5%)	
Stem type, n (%)				**<.001**
2: Single wedge	294 (38.0%)	271 (37.6%)	23 (43.4%)	
3: Double wedge	116 (15.0%)	99 (13.7%)	17 (32.1%)	
4A: Triple-taper metaphyseal	311 (40.2%)	305 (42.3%)	6 (11.3%)	
4B: Gradual taper/metadiaphyseal filling	53 (6.8%)	46 (6.4%)	7 (13.2%)	

SD, standard deviation.

Among 774 THA cases, PFF occurred in 53 patients (6.8%) within 90 days including 23 intraoperative fractures and 30 postoperative PFF. Fifteen patients required reoperation. Average time to fracture was 4.7 ± 2.8 weeks. Patients were older (mean age 70.5 vs 65.1 years, *P* < .001) and more likely to be female (73.6% vs 56.0%, *P* = .02) comparing those who sustained a PFF to no fracture. In the triple-taper metaphyseal group, 6 periprosthetic fractures occurred postoperatively including four patients who reported a mechanical fall mechanism, one who complained of persistent pain and was diagnosed with a PFF and 1 patient who felt a “pop” and was diagnosed with a fracture. The incidence of early PFF varied significantly by stem design: triple-taper metaphyseal stems had the lowest rate (1.9%), followed by single-wedge (7.8%), gradual taper/metadiaphyseal (13.2%), and double-wedge stems (14.7%) (*P* < .001) (Table 3). This protective effect was consistent across Dorr A and B subtypes and across age groups (Table 3). In the multivariable regression analysis, after adjusting for patient age and sex, triple-taper metaphyseal stems were associated with a decreased risk of early PFF compared to all other designs (OR 0.20; 95% CI 0.08-0.48; *P* < .001). Among collared stems, PFF occurred in 6 of 311 (1.9%) triple-taper metaphyseal stems compared with 7 of 53 (13.2%) gradual taper/metadiaphyseal-filling stems. The gradual taper/metadiaphyseal-filling stem was associated with significantly greater odds of PFF than the triple-taper metaphyseal stem (OR 7.74; 95% CI, 2.49-24.03; *P* < .001) (Table 4). Within the triple-taper metaphyseal stem group, the collar was positioned on the calcar in 248 patients and was proud in 63 patients (23.3% proud) (Figure 1). The PFF risk was significantly increased if the calcar was proud and not seated on the calcar (6.3% vs 0.8%, respectively; *P* = .02) (Table 5).

**Table 3:** PFF risk by stem design, age, and Dorr classification.

Variable	Non–triple-taper metaphyseal	Triple-taper metaphyseal	OR (95% CI)	*P* value	Single wedge	Triple-taper metaphyseal	OR (95% CI)	*P* value	Double wedge	Triple-taper metaphyseal	OR (95% CI)	*P* value	Gradual taper/metadiaphyseal filling	Triple-taper metaphyseal	OR (95% CI)	*P* value
Age <65 yo	13 (7.5%)	3 (2.0%)	**4.00 (1.07**-**22.29)**	**.037**	5 (4.1%)	3 (2.0%)	**2.12** (**0.40**-**13.91)**	**.473**	5 (14.3%)	3 (2.0%)	**8.14 (1.49**-**55.23)**	**.007**	3 (17.6%)	3 (2.0%)	**10.34 (1.27**-**84.77)**	**.014**
Age 65-75 yo	14 (8.0%)	3 (2.3%)	**3.64 (0.99**-**20.17)**	**.042**	7 (7.0%)	3 (2.3%)	**3.15** (**0.70**-**19.35)**	**.108**	6 (11.3%)	3 (2.3%)	**5.30 (1.08**-**34.09)**	**.019**	1 (4.5%)	3 (2.3%)	**1.99 (0.04**-**26.14)**	**.471**
Age >75 yo	20 (17.5%)	0 (0%)	**∞ (1.45**-**∞)**	**.014**	11 (15.3%)	0 (0%)	**∞** (**1.13**-**∞)**	**.031**	6 (21.4%)	0 (0%)	**∞ (1.41**-**∞)**	**.009**	3 (21.4%)	0 (0%)	**∞ (0.96**-**∞)**	**.028**
Dorr A	27 (8.0%)	3 (1.8%)	**4.69 (1.41**-**24.53)**	**.005**	13 (6.0%)	3 (1.8%)	**3.43** (**0.92**-**19.09)**	**.068**	9 (11.0%)	3 (1.8%)	**6.60 (1.59**-**39.01)**	**.003**	5 (13.2%)	3 (1.8%)	**8.06 (1.49–54.41)**	**.007**
Dorr B	20 (15.9%)	1 (1.0%)	**19.27 (2.97**-**813.13)**	**<.001**	10 (13.0%)	1 (1.0%)	**15.18** (**2.08**-**673.45)**	**<.001**	8 (23.5%)	1 (1.0%)	**30.71 (3.84**-**1419.19)**	**<.001**	2 (13.3%)	1 (1.0%)	**15.15 (0.74–943.32)**	**.042**

**Table 4:** PFF risk according to femoral stem design and collar presence.

Stem design	Implant(s)	Collar	N	PFF, n (%)	OR (95% CI)	*P* value
Triple-taper metaphyseal	Insignia	Yes	311	6 (1.9)	Reference	—
Gradual taper/metadiaphyseal-filling	Polarstem	Yes	53	7 (13.2)	7.74 (2.49-24.03)	**<.001**

**Figure 1:**
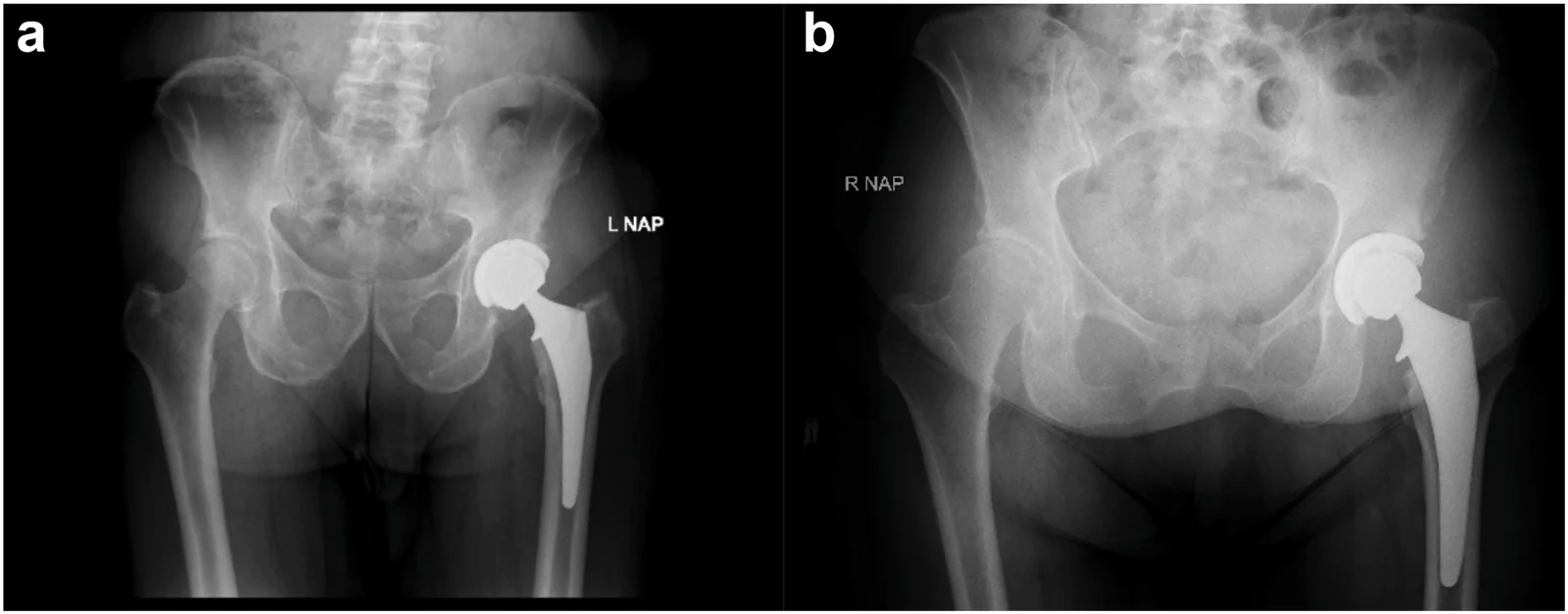
Representative postoperative radiographs demonstrating femoral stem collar position. (a) Postoperative radiograph demonstrating appropriate collar seating with the femoral stem collar in direct contact with the calcar. (b) Postoperative radiograph demonstrating a proud collar, with a visible gap between the femoral stem collar and the calcar.

**Table 5:** PFF risk according to collar position in triple-taper metaphyseal stems.

Collar position	N	PFF, n (%)	No PFF, n (%)	OR (95% CI)	*P* value
Collar on the calcar	248	2 (0.8)	246 (99.2)	Reference	—
Collar above the calcar	63	4 (6.3)	59 (93.7)	8.34 (1.49-46.62)	**.02**

## Discussion

This study reinforces the critical role of femoral stem design in reducing early periprosthetic fracture following cementless THA. Our findings demonstrate that triple-taper, metaphyseal-engaging stems are associated with a significantly lower incidence of early PFFs than single-wedge, double-wedge, and gradual taper/metadiaphyseal-filling designs across age and bone quality profiles. Additionally, collar positioning on the calcar further lowers PFF incidence.

Patient factors such as older age, female sex, and poor bone quality have been established as independent risk factors for PFF after THA [14], [15], [16]. Prior studies have shown nearly threefold increased fracture risk in patients over 70 years old [15], nearly double the odds in women vs men [14], and over fivefold increased risk in patients with Dorr type C femurs compared to Dorr B [16]. Recognizing these risk factors is essential, but equally important is understanding how fixation method and implant design interact to influence outcomes. Traditionally, cemented femoral fixation remains one of the most reliable techniques for achieving stable fixation, demonstrating consistently lower fracture rates than uncemented femoral stems [7], [17], [18], [19], [20], [21], [22], [23], particularly in elderly patients and those with compromised bone stock. In a comprehensive 40-year review from a single institution, Abdel et al reported that the incidence of postoperative femoral fracture within 30 days of primary THA was ten times higher in uncemented stems, independent of patient age or sex. Further, intraoperative fracture rates were significantly lower with cemented fixation (0.2%) vs uncemented stems (3.0%) [7]. Further, in a propensity score matched analysis of patients aged 65 years and older undergoing THA for femoral neck fractures, Hameed et al. found that cemented fixation (n = 1124) was associated with a lower rate of periprosthetic fracture than cementless fixation (n = 2842) (2.5% vs 4.4%; *P* = .02) [20]. More recently, Neitzke et al. evaluated a modern triple-tapered, noncemented, collared stem using American Joint Replacement Registry data in patients older than 65 years. The authors reported a threefold lower 2-year PFF incidence than aggregated noncemented collarless stems (0.19% vs 0.65%, *P* < .001), with a fracture incidence comparable to cemented stems (0.19% vs 0.20%, *P* = .99) [11]. Our findings are consistent with these registry data while providing a more granular comparison of specific cementless stem geometries. We found that triple-taper metaphyseal stems had a significantly lower 3-month PFF incidence than single-wedge, double-wedge, and gradual taper/metadiaphyseal-filling designs. Additionally, our analysis incorporated proximal femoral morphology and collar position, demonstrating that a collar seated on the calcar was associated with a lower PFF incidence than a proud collar. Together, these findings further support the protective role of contemporary triple-tapered, collared stem designs and suggest that both stem geometry and optimal collar-calcar contact may contribute to reducing early PFF risk.

Our results reinforce the published literature demonstrating the influence of stem geometry and collar design on PFF risk [10], [24], [25]. In an analysis of the American Joint Replacement Registry, Kelly et al. reported that single- and double-wedge stems were associated with a nearly threefold increased risk of PFF in patients aged 65 and older, compared to gradual taper/metadiaphyseal-filling stems (Hazard Ratio [HR] 2.9, 95% CI 2.2-3.9 and HR 3.0, 95% CI 2.2-4.0, respectively; *P* < .001) [10]. Similarly, a retrospective review of 3183 posterior-approach THAs by Radaelli et al [26] classified stems into single-wedge (type 1), double-wedge (type 2), and ream-and-broach (type 3) configurations. Type 2 stems demonstrated a significantly higher rate of all-cause PFFs (3.6%) than both the type 1 (0.7%) and type 3 (0.4%) designs (both *P* < .01) [27]. Further supporting the mechanical basis of early fractures, a multicenter retrospective analysis of 3433 cementless THAs compared single-taper, double-taper, and compaction-collared stems (Corail (DePuy; Raynham, MA). The compaction-collared group exhibited significantly lower rates of Vancouver B-type fractures (HR 0.18, 95% CI 0.03-0.97; *P* = .044) with a median time to fracture of just 8.8 weeks and a reoperation rate of 4.8% [25]. These findings underscore the early, mechanically driven nature of many PFFs and the potential protective role of compaction-based implant techniques.

Triple-taper stems were not included in these prior comparative studies, and inconsistent classification systems across the literature have made it difficult to evaluate modern implant designs directly. Our study expands on the work presented by Herndon et al. [9] by including a large cohort of triple-taper stems and applying an updated classification framework that better reflects contemporary stem geometry. By doing so, we not only confirm a lower fracture risk associated with triple-taper stems, showing a significantly reduced hazard of periprosthetic fracture (HR 0.21, 95% CI 0.09-0.49, *P* < .001), but also suggest these stems may offer even greater protection against early PFF across variations in age, sex, and bone quality. A recent cohort study of over 23,000 primary cementless THA cases from a single-institution joint registry database reported that femoral stem morphology significantly influenced the risk of PFF [28]. Among the four stem designs evaluated—single-taper, dual-taper, quadrangular-taper, and triple-taper collared—the triple-taper collared stems demonstrated the lowest rates of both intraoperative and early postoperative fractures (0.37%). These associations remained statistically significant after adjusting for demographic and clinical variables, suggesting an independent protective effect [28]. Additionally, collared stems as a group were associated with lower rates of early postoperative fractures than noncollared designs [28]. These findings are consistent with the results of our study, which also identified triple-taper stems as having the lowest overall PFF risk among contemporary cementless designs after multivariable adjustment highlighting its advantage over both wedge-based and other tapering stem geometries. Our cohort study expands on that of LeBrun et al. [28], by also assessing Dorr and collar positioning allowing for a more detailed exploration of how stem design relates to patient-specific and surgical factors.

In additional to stem geometry, the presence of a collar has also been shown to be protective of PFF [10], [29]. In a large retrospective review of 11,522 primary THAs in patients over age 65, Rodriguez et al. found that noncollared cementless stems were associated with significantly higher odds of early PFF than collared cementless stems (OR 3.11; *P* = .03) [29]. Similarly, Kelly et al. reported that collarless metadiaphyseal-filling stems had an elevated risk of all-time PFF compared to their collared counterparts (HR 7.8, 95% CI 4.1-15; *P* < .001) [10]. Importantly, the lower PFF incidence observed with the triple-taper metaphyseal stem in our study does not appear to be explained by collar presence alone. When the analysis was restricted to collared stems, the triple-taper metaphyseal design had a PFF incidence of 1.9% compared with 13.2% for the collared gradual taper/metadiaphyseal-filling design. The latter was associated with more than sevenfold greater odds of PFF than the triple-taper metaphyseal stem. Although this subgroup comparison was unadjusted and should be interpreted cautiously, it suggests that additional implant characteristics, including stem geometry and the manner in which the stem engages the proximal femur, may contribute to early fracture risk beyond the presence of a collar itself. Furthermore, our findings suggest that the potential benefit of a collar may depend not only on its presence but also on its position relative to the calcar. Within the triple-taper metaphyseal cohort, stems with the collar seated on the calcar demonstrated a significantly lower PFF incidence than stems with a proud collar (0.8% vs 6.3%; *P* = .02).This finding supports cadaveric biomechanical studies demonstrating that optimal collar seating improves load transfer and reduces anterior and medial cortical strain and may avoid femoral component subsidence in the early postoperative period [30], [31].

The advantageous performance of triple-taper metaphyseal stems likely results from a combination of design and surgical factors. These stems are typically implanted using a broach-only technique, which compacts on the anterior and posterior portions of the femoral canal and removes bone medially and laterally, helping preserve and strengthen trabecular structure. Their gradual taper in three-dimensions allows for stable engagement of both the metaphyseal and diaphyseal regions, promoting improved fixation and more even stress distribution. The stems also feature a size-specific medial curvature and a trapezoidal cross-section, which enhances proximal canal fill in both the coronal and sagittal planes. This design more closely mirrors native femoral anatomy, potentially reducing micromotion and improving rotational and implant stability. Additionally, the inclusion of a size-matched collar is intended to optimize calcar contact, which may further support mechanical integrity. We encourage surgeons to consider these modern triple-taper metaphyseal stems with a collar seated on the calcar given their protection against periprosthetic femur fracture in the early postoperative period.

This study has several limitations. Implant selection in the comparison group was not randomized and was left to surgeon discretion, introducing potential selection bias. Each stem design category included multiple implant models, and small differences in design or material properties may affect outcomes. To better reflect the spectrum of modern implants, we applied an updated stem classification system. This allowed us to more accurately compare contemporary designs and capture clinically relevant distinctions between stems that would be grouped together using legacy schemas. We did not assess changes in implant preference over time, which may introduce confounding. Furthermore, given the multicenter nature of the study, there was variability in surgical technique and perioperative protocols. Although this enhances generalizability, it also introduces heterogeneity that we could not fully account for in this study. Additionally, we did not control for all patient- or procedure-specific factors such as surgical approach, BMI, or comorbidities. Our cohort, drawn from a tertiary referral center, may also overrepresent complex cases. Prospective, randomized studies are warranted to validate our findings and to better define the optimal implant characteristics for reducing early mechanical failure in cementless THA.

## Conclusions

We found that femoral stem geometry is a key determinant of early periprosthetic femur fracture risk in cementless THA. The triple-taper metaphyseal stem significantly reduces fracture incidence across a range of patient ages and bone qualities. Ensuring that the stem collar is seated on the calcar further decreases the periprosthetic fracture risk. Based on these results, we recommend preferential selection of this stem design in cementless primary THA, especially for patients at increased risk of PFF due to advanced age, female sex, or bone quality.

## CRediT authorship contribution statement

**Alexandra N. Krez:** Writing – original draft, Investigation, Formal analysis, Data curation. **Sean D. Toomey:** Writing – review & editing, Conceptualization. **Kyle C. Swanson:** Writing – review & editing, Conceptualization. **Carl L. Herndon:** Writing – review & editing, Conceptualization. **Jonathan R. Danoff:** Writing – review & editing, Supervision, Project administration, Methodology, Data curation, Conceptualization.

## Conflict of interest

Sean D. Toomey is a paid consultant for Stryker; Becton Dickinson; receives research support from Stryker. Kyle C. Swanson is a paid consultant for Stryker and recives royalties from Exactech; Jonathan R. Danoff is a paid consultant for Stryker; Corza Medical; Microgendx; Sanara Medtech; receives research support from Stryker; serves on the editorial/governing board for Arthroplasty Today; serves as a board or committee member for AAHKS Committee. Carl L. Herndon is a paid consultant for Depuy; serves on the editorial board for JOA; serves as a board or committee member for Advocacy Committee, AAHKS. All other authors declare no potential conflicts of interest.

For full disclosure statements refer to https://doi.org/10.1016/j.artd.2026.102193.

## Appendix A Supplementary data

Conflict of Interest Statement for Krez

Conflict of Interest Statement for Herndon

Conflict of Interest Statement for Danoff

Conflict of Interest Statement for Swanson

Conflict of Interest Statement for Toomey
