# Supplementary material for: Femoral Stem Design Influences Early Periprosthetic Fracture Risk Following Total Hip Arthroplasty
Source: Arthroplast Today. 2026 Oct 31;42:102193. doi: 10.1016/j.artd.2026.102193 (PMC13744306; doi:10.1016/j.artd.2026.102193)
Supplement: Supplementary file 1 [file mmc1.pdf]

# CONFLICT OF INTEREST STATEMENT

*The Journal of Arthroplasty*

(Adopted from the American Academy of Orthopaedic Surgeons disclosure statement)

The following form **must be filled out completely and submitted by each author (example, 6 authors, 6 forms). If no discloser is required, please write/type "none" at the end of each sentence**.

---

Manuscript Title Femoral Stem Design Influences Early Periprosthetic Fracture Risk Following Total Hip Arthroplasty

1. Royalties from a company or supplier (The following conflicts were disclosed)
None
2. Speakers bureau/paid presentations for a company or supplier (The following conflicts were disclosed)
None

3A. Paid employee for a company or supplier (The following conflicts were disclosed)
None

3B. Paid consultant for a company or supplier (The following conflicts were disclosed)
None

3C. Unpaid consultants for a company or supplier (The following conflicts were disclosed)
None

4. Stock or stock options in a company or supplier (The following conflicts were disclosed)
None
5. Research support from a company or supplier as a Principal Investigator (The following conflicts were disclosed)
None
6. Other financial or material support from a company or supplier (The following conflicts were disclosed)
None
7. Royalties, financial or material support from publishers (The following conflicts were disclosed)
None
8. Medical/Orthopaedic publications editorial/governing board (The following conflicts were disclosed)
None
9. Board member/committee appointments for a society (The following conflicts were disclosed)
None

**Each author must sign AND print or type his/her name, date and submit a separate form.**

In addition, one BLINDED Conflict of Interest form (no author names used) should be submitted per manuscript with all author disclosures.

| Alexandra Krez | Alexandra Krez | 02/11/2026 |
| --- | --- | --- |
| Author Name (Print or Type) | Author Signature | Date |
