# Supplementary material for: Femoral Stem Design Influences Early Periprosthetic Fracture Risk Following Total Hip Arthroplasty
Source: Arthroplast Today. 2026 Oct 31;42:102193. doi: 10.1016/j.artd.2026.102193 (PMC13744306; doi:10.1016/j.artd.2026.102193)
Supplement: Supplementary file 2 [file mmc2.pdf]

INDIVIDUAL CONFLICT OF INTEREST STATEMENT

American Association of Hip and Knee Surgeons

(Adopted from the American Academy of Orthopaedic Surgeons disclosure statement)

The following form **must be filled out completely and submitted by each author (example, 6 authors, 6 forms). All items require a response. If there is no relevant disclosure for a given item, enter "*None.*"**

Manuscript Title: Femoral Stem Design Influences Early Periprosthetic Fracture Risk Following Total Hip Arthroplasty

1. Royalties from a company or supplier (The following conflicts were disclosed)

No

2. Speakers bureau/paid presentations for a company or supplier (The following conflicts were disclosed)

No

3A. Paid employee for a company or supplier (The following conflicts were disclosed)

No

3B. Paid consultant for a company or supplier (The following conflicts were disclosed)

Depuy

3C. Unpaid consultants for a company or supplier (The following conflicts were disclosed)

No

4. Stock or stock options in a company or supplier (The following conflicts were disclosed)

No

5. Research support from a company or supplier as a Principal Investigator (The following conflicts were disclosed)

No

6. Other financial or material support from a company or supplier (The following conflicts were disclosed)

No

7. Royalties, financial or material support from publishers (The following conflicts were disclosed)

No

8. Medical/Orthopaedic publications editorial/governing board (The following conflicts were disclosed)

Editorial Board, JOA

9. Board member/committee appointments for a society (The following conflicts were disclosed)

Advocacy Committee, AAHKS

Each author must sign AND print or type his/her name, date and submit a separate form

In addition, one BLINDED Conflict of Interest form (no author names used) should be submitted per manuscript with all author disclosures.

Carl L. Herndon	*Carl Herndon*	01/28/2026
Author Name (Print or Type)	Author Signature	Date
